# A Clinician and Electronic Health Record Wearable Device Intervention to Increase Physical Activity in Patients With Obesity: Formative Qualitative Study

**DOI:** 10.2196/56962

**Published:** 2024-09-02

**Authors:** Varun Ayyaswami, Jeevarathna Subramanian, Jenna Nickerson, Stephen Erban, Nina Rosano, David D McManus, Ben S Gerber, Jamie M Faro

**Affiliations:** 1 Department of Medicine University of Massachusetts Chan Medical School Worcester, MA United States; 2 Department of Population and Quantitative Health Sciences University of Massachusetts Chan Medical School Worcester, MA United States

**Keywords:** remote patient monitoring, physical activity, electronic health record, wearable device, patient monitoring, health monitoring, health monitor, patient monitor, remote patient monitor, exercise, exercises, electronic health records, patient record, health record, health records, wearable devices

## Abstract

**Background:**

The number of individuals using digital health devices has grown in recent years. A higher rate of use in patients suggests that primary care providers (PCPs) may be able to leverage these tools to effectively guide and monitor physical activity (PA) for their patients. Despite evidence that remote patient monitoring (RPM) may enhance obesity interventions, few primary care practices have implemented programs that use commercial digital health tools to promote health or reduce complications of the disease.

**Objective:**

This formative study aimed to assess the perceptions, needs, and challenges of implementation of an electronic health record (EHR)–integrated RPM program using wearable devices to promote patient PA at a large urban primary care practice to prepare for future intervention.

**Methods:**

Our team identified existing workflows to upload wearable data to the EHR (Epic Systems), which included direct Fitbit (Google) integration that allowed for patient PA data to be uploaded to the EHR. We identified pictorial job aids describing the clinical workflow to PCPs. We then performed semistructured interviews with PCPs (n=10) and patients with obesity (n=8) at a large urban primary care clinic regarding their preferences and barriers to the program. We presented previously developed pictorial aids with instructions for (1) providers to complete an order set, set step-count goals, and receive feedback and (2) patients to set up their wearable devices and connect them to their patient portal account. We used rapid qualitative analysis during and after the interviews to code and develop key themes for both patients and providers that addressed our research objective.

**Results:**

In total, 3 themes were identified from provider interviews: (1) providers’ knowledge of PA prescription is focused on general guidelines with limited knowledge on how to tailor guidance to patients, (2) providers were open to receiving PA data but were worried about being overburdened by additional patient data, and (3) providers were concerned about patients being able to equitably access and participate in digital health interventions. In addition, 3 themes were also identified from patient interviews: (1) patients received limited or nonspecific guidance regarding PA from providers and other resources, (2) patients want to share exercise metrics with the health care team and receive tailored PA guidance at regular intervals, and (3) patients need written resources to support setting up an RPM program with access to live assistance on an as-needed basis.

**Conclusions:**

Implementation of an EHR-based RPM program and associated workflow is acceptable to PCPs and patients but will require attention to provider concerns of added burdensome patient data and patient concerns of receiving tailored PA guidance. Our ongoing work will pilot the RPM program and evaluate feasibility and acceptability within a primary care setting.

## Introduction

Limited physical activity (PA) is a modifiable risk factor associated with obesity [1]. Obesity is strongly associated with numerous medical comorbidities, as well as increased all-cause mortality, health care use, and expenditure per capita [2-5]. Lifestyle modifications incorporating increased PA in the comprehensive management of obesity improve both weight loss and the prevention of weight gain [6,7]. Furthermore, patients with obesity can improve cardiometabolic health, cardiorespiratory fitness, and muscle strength through increased PA independent of weight loss [8]. With 75% of the current US population with overweight or obesity [9], increasing PA has been a critical public health effort supported by the US Preventive Services Task Force clinical guidelines [10,11]. Lifestyle medicine counseling by physicians during routine care promoted clinically significant weight loss, increased patient motivation and PA, and improved diet [12,13]. However, counseling in primary care settings is infrequent, with less than 20% of patients reporting receiving weight counseling [14,15].

The number of individuals using commercial digital health devices has grown in recent years to more than 1 in 5 Americans [16,17]. These higher rates of use in patients suggest a potential tool that primary care providers (PCPs) can leverage to improve PA counseling for their patients. Notably, a meta-analysis of remote patient monitoring (RPM) showed that successful RPM PA interventions for weight loss specifically incorporated tailored health coaching or other behavior change models [18]. However, the implementation of digital tools into clinical workflow in the primary care setting is limited by a lack of end-to-end support for patients and PCPs to appropriately leverage these tools for obesity management. Specifically, common barriers to health care provider adoption of patient-collected data include challenges of wearable device integration into the electronic health record (EHR), maintenance of privacy and confidentiality of patient data, lack of system interoperability and connectivity of wearable devices and health systems, and patient information or data overload [19].

We evaluated the planned implementation of an existing wearable device integration into the EHR (Epic Systems) at an urban primary care practice. The purpose of this formative study was to assess the perceptions, barriers, and challenges of implementing an EHR-integrated RPM program using a wearable device to promote patient PA at a large urban primary care practice through patient and provider interviews.

## Methods

### Overview

First, we identified and described existing clinical workflows. Then, we conducted semistructured interviews with patients and providers to identify the perceptions, needs, and challenges of implementing an EHR-integrated RPM program within these workflows to prepare for a future intervention. No clinical intervention was performed in this study. This study followed applicable Standards for Reporting Implementation Studies (StaRI) guidelines (Multimedia Appendix 1).

### Ethical Considerations

The study was approved by the University of Massachusetts (UMass) Chan Institutional Review Board (protocol #00000781) on January 17, 2023. Provider participants were provided a fact sheet and gave verbal consent before interviews. Patient participants gave written informed consent before interviews. All participants received compensation in the form of a US $50 gift card after completion of interviews. All study data were deidentified.

### Clinical Workflows

First, our team explored available options to upload commercial wearable device data for PA to the EHR. We discovered a “fitness device flowsheet” order is available within the EHR. This order allowed providers to request patient fitness device metrics such as steps to sync with the EHR as a flowsheet, facilitating a display of device metrics within the EHR, similar to the display of vital sign data (ie, blood pressure). We also found a Fitbit integration available to sync patient PA data from the Fitbit app to the patient portal (myChart) after the flowsheet was ordered by a provider. The ordering provider is then able to review patient data through the EHR through basket message notification. In addition, any provider can view the patient’s PA data in the patient chart. We identified an existing EHR job aid that could be used to educate providers on ordering the fitness device flowsheet (Multimedia Appendix 2). The EHR job aid included details on types of patient data (ie, steps) that could be integrated into the EHR and information on the provider clinical workflow process to set up and receive this data. We also found instructions showing patients how to connect mobile and wearable devices to the patient portal (Multimedia Appendix 3). Although we identified multiple workflow steps necessary for an RPM program within our clinical system, no clinicians were using any of the steps regularly and in a cohesive workflow manner. For example, our review indicated that within the 1-year period before this study (from March 31, 2022, to February 28, 2023), the fitness device flowsheet was ordered a total of 4 times by all providers employed by UMass Memorial Health.

### Provider Participant Recruitment and Interviews

In April 2023, we recruited 10 PCPs from the UMass Chan Division of General Internal Medicine to participate in the study. Our recruited sample size was selected to reach saturation point and consistent with other qualitative studies noting that saturation can be reached with a relatively low sample size [20]. We aimed to recruit at least 1 attending physician, resident physician, and advanced practitioner provider. Providers were recruited by email and at a study presentation at a monthly clinic meeting. Providers were eligible if they were (1) an attending physician, resident physician, or advanced practitioner provider practicing in the UMass Memorial Medical Group Primary Care Clinic; (2) older than 18 years of age; and (3) spoke English.

Providers took part in a 30-minute semistructured interview led by a study team member. They were interviewed by a study team member to assess perceptions, needs, and challenges of implementation of an EHR-integrated RPM program. Participant interview questions are available in Multimedia Appendix 4. During the interview, we shared the pre-existing EHR job aid used to train providers on ordering the fitness device flowsheet (Multimedia Appendix 1) and obtained feedback on the existing clinical workflow.

### Patient Participant Recruitment and Interviews

From May to September 2023, we recruited 8 patients from the UMass Memorial Medical Group Primary Care Clinic to participate in the study. As with provider interviews, our sample size was selected to reach saturation point and consistent with other qualitative studies [20]. We aimed to recruit at least 1 patient identifying as African American and 1 patient identifying as Hispanic. Patients were recruited from the clinical practice of JS during routine clinical visits. Patients who expressed interest in learning more about the study were referred to meet with a study research coordinator. Patients were eligible to participate if they (1) received care at a UMass Memorial Primary Care Clinic and (2) had a BMI of 30 kg/m^2^ or greater (also verified through self-report). Patients were ineligible if they were (1) unable to provide informed consent, (2) incarcerated, (3) were non-English speaking, or (4) were younger than 18 years of age.

Patients completed semistructured interviews regarding their preferences for remote monitoring, PA counseling, data-sharing with providers, and enrollment processes. Participant interview questions are found in Multimedia Appendix 5. We also showed patients screenshots from myChart to obtain feedback on the existing patient-facing workflow.

### Analysis

All interviews were transcribed and reviewed for clarity. We used rapid qualitative analysis during and after the interviews [21,22]. We used an Excel spreadsheet (Microsoft Corporation) to capture data, with each participant as a row, and domains of interest as columns. The coding team (JF, JN, JS, and VA) met initially to develop key domains that addressed our research question and were derived from the interview guides. The team then conducted 2 rounds of coding together, before independently coding an additional 2 transcripts per coding team member. Once all the transcripts were coded, JF and VA independently reviewed the results of each column to identify themes within each domain of interest. They then reviewed across columns to identify any overlap in themes across the domains. Both coders had a high agreement on themes after this process, determined saturation had been met with codes, and reached a consensus on the final list of themes. The team used a validated checklist to ensure interrater reliability [23].

## Results

### Participants

The sample included 10 provider participants, 50% (5/10) of which were female and the rest 50% (5/10) were male. In all, 40% (4/10) were residents, 20% (2/10) were providers, and 40% (4/10) were attendings, with 40% (4/10) having 0-3 years of experience, 20% (2/10) having 3-9 years of experience, and 40% (4/10) having 10+ years of experience (Table 1). We also included 8 patient participants: 88% (7/8) were female and 12% (1/8) were male, with a mean age of 58.5 (SD 13) years. Of these participants, 25% (2/8) were non-White, 75% (6/8) were White, 88% (7/8) were non-Hispanic, and 12% (1/8) were Hispanic.

**Table 1:** Characteristics of provider (n=10) and patient (n=8) participants who completed interviews to assess implementing an electronic health record–integrated remote patient monitoring program using a wearable device to promote physical activity for patients with obesity.

Participants				Values
**Provider participants (n=10)**				
	**Sex, n (%)**			
		Female	5 (50)	
		Male	5 (50)	
	**Type of provider, n (%)**			
		Internal medicine resident	4 (40)	
		Advanced practice provider	2 (20)	
		Attending physician	4 (40)	
	**Years of experience, n (%)**			
		0-3	4 (40)	
		3-9	2 (20)	
		10 or more	4 (40)	
**Patient participants (n=8)**				
	**Gender, n (%)**			
		Female	7 (88)	
		Male	1 (12)	
	Age (years), mean (SD)			58.5 (13)
	**Race, n (%)**			
		African American or Black	1 (12)	
		White	6 (75)	
		Other	1 (12)	
	**Ethnicity, n (%)**			
		Hispanic or Latino	1 (12)	
		Non-Hispanic or Latino	7 (88)	

### Provider Participant Feedback

Provider feedback regarding the implementation of an RPM program around PA using commercial wearables was positive. Providers appreciated the importance of PA guidance for healthy weight maintenance. They noted the proposed clinical workflow appeared simple to implement in practice. However, potential challenges were noted related to provider burden, patient and provider resource support, and health equity. Overall, 3 themes were identified from the interviews: (1) providers’ knowledge of PA prescription is focused on general guidelines with limited knowledge on how to tailor guidance to patients, (2) providers were open to receiving PA data but were worried about being overburdened in their workflow, and (3) providers were concerned about patients being able to equitably access and participate in digital health interventions. Themes and representative quotes are included in Textbox 1.

**Provider participant themes and representative quotes from semistructured interviews (n=10) to assess implementing an electronic health record–integrated remote patient monitoring program using a wearable device to promote physical activity for patients with obesity.**

**Theme 1**

- Focus on general physical activity guidelines with limited knowledge on how to modify guidance for patient-specific factors.
  - “My general recommendations is 30 to 45 minutes of cardiovascular activity most days of the week. I’m not an exercise physiologist, so I don't get too in the weeds...”
  - “I don’t specifically prescribe certain types of exercise. I don’t have any particular knowledge other than my own physical therapy experience. I can talk to people about gradually working into their exercise and working up gradually. I don’t tend to get into specifics of types.”
  - “I haven’t really had too much like training in it specifically, but I have a strong interest in exercise and fitness and everything in general. I suggest starting really low with basic type of things. But when it comes to patients that have trouble walking or doing exercises because of pain, I have a really hard time with helping them because I don't know what to suggest”

**Theme 2**

- Receptive to receiving physical activity data but were worried about being overburdened by additional work.
  - “I can see the utility in this. Somebody’s keeping an eye on It, they may be more likely to follow through with those recommendations, if they buy in. I can see it being a quick thing that you could send a note to the patient and either encourage them or congratulate ‘em on how they’re doing. The concern with it is that the inbox is already cluttered. Depending on the frequency of it, it becomes one more thing that you need to deal with.”
  - “That’s cool. It seems to be easy enough to set up. I think to be able to view the data is good but I think your next step to actually help the clinical team would be if Epic can automatically graph <the data>. Getting the raw information is unlikely to be very helpful if the provider has limited time to review it...you do want to have that ability to analyze the data very quickly”
  - “Having that order set is great...what I fear about <getting notification in> myChart messages is that it is just more work to do. I like the fact that you’re able to identify how often you get the alerts because I can just time it for another visit in one month”

**Theme 3**

- Concerned about patients being able to equitably access and participate in digital health interventions.
  - “I’d wanna be sure that I wasn’t prescribing something to patients that would cause them a lot of financial burden. I’m not even sure if this is covered by insurance. Is this something that I would be sticking my patient with a $200 device when they only make a few hundred bucks a week?”
  - “I would also think about age as a major barrier for some folks...I don’t think that would be as good of a fit for some older folks who struggle with technology...what I’m not sure of is whether or not people from different backgrounds would be equally receptive to having a wearable, trackable thing.”
  - “I’m just thinking there’s logistical and equity issues with our patients that became very obvious during the pandemic where we were trying to do telehealth with everybody and so many people just didn't have the technology to do that.”

### Patient Participant Feedback

Patient participant feedback from interviews regarding RPM of PA was enthusiastic. The enthusiasm stemmed from their frustration about perceived inadequate PA guidance from their health care team. The patient noted previous positive experiences with wearable devices but noted inconsistent adherence without external reinforcement. They were in favor of sharing exercise metrics with providers to facilitate improved counseling from the health care team. They did not foresee significant difficulty in setting up an RPM program from an IT perspective if specific support was provided. A total of 3 themes were identified from the analysis, including (1) patients received limited or general guidance regarding PA from providers and other resources, (2) patients want to share exercise metrics with the health care team and receive tailored PA guidance at regular intervals, and (3) patients need written resources to support the setting up of a wearable program with access to live supports on an as-needed basis. Themes and representative quotes are included in Textbox 2.

**Patient participant themes and representative quotes from semistructured interviews (n=8) to assess the implementation of an electronic health record–integrated remote patient monitoring program using a wearable device to promote physical activity for patients with obesity.**

**Theme 1**

- Receive limited or general guidance regarding physical activity from providers and other resources.
  - “She asked me what I did. I just walk. That’s about all my activity besides what I do at work, which is walking as well. I don’t usually [get physical activity guidance from any resource]”
  - “Just to work out, make sure that I'm exercising. My doctor, she’s great. She just said 210 minutes of activity a week, which I pretty much get. I don’t really get advice from anyone else.”
  - “Just your basic, what your doctors tells you, try to meet the 10,000 steps. Heart health, focus on just meeting that normal every day. What you hear on tv, what you see online. It’s everywhere. It’s always in your face if you're paying attention. But when your doctors tell you try to meet that 10,000 steps, try to walk instead, park far away and walk into the store, just get extra steps in.

**Theme 2**

- Want to share exercise metrics with the health care team and receive tailored physical activity guidance at regular intervals.
  - “I think it would be a good tool [to share physical activity metrics]...So I think for me, kind of busy life working children, which we all have. I think weekly [check ins with health care team] would kind of be overkill. Um, you know, I think monthly I would be okay with anything longer than that...But again, I think it's also patient specific, Like depending on where they're at too. Some, some patients may need a weekly check-in until they get to a certain point”
  - “I’ll share it with the whole world. I just want them to figure out what's going on. So if it’s something that can help you and you’re honest with your doctors you shouldn’t be afraid to share.”
  - “I wanna know, okay, what exercises can I do? What can I do to calm the nerve, break it up and relax me so that I can actually physically get out of my bed and function? So if there was more information on that, that would be phenomenal. The best exercises for back pain, hip pain, certain age groups...my age group is different than a 20 year old.”

**Theme 3**

- Need written resources to support the setting up of a wearable program with access to live support on an as-needed basis.
  - “I think that’s very patient specific just because there’s all different levels out there, where some people may have no idea and it may be their first time or some people are a little tech savvy and they'd be okay with it. I’d be okay even again, just with a printout of, hey, these are the steps and you know, if you need help or whatever, then give us a call...I think a Zoom would be fine for myself [if clarifications were needed after written instructions].”
  - “If the word for word instructions were clear, I could do that...[also] I just, I, I, I like having the feedback a little better than the printed stuff. ‘cause sometimes, you know, you, you think you understand what the, what the instructions are saying, and sometimes you don’t..., however it comes, as long as the instructions are clear, I’m fine.”
  - “I have to say. I’m not fabulous with computers and whatnot. But I am on a computer now every day...Usually I can follow directions pretty well. If it’s online, I can just walk myself through it. Just written instructions really. And I can pretty much do it or anything.”

## Discussion

### Principal Findings

Our preliminary study assessed the perceptions of, need for, and the challenges of implementing an EHR-integrated RPM program to facilitate PA counseling of primary care patients through patient and provider interviews. We found that providers had limited confidence in their abilities to tailor PA recommendations to patient-specific clinical factors. Similarly, patients felt PA counseling received from their health care team was not individualized to factors such as age and medical conditions. However, patients were eager to share PA data through an EHR integration in the hopes it could facilitate more specific PA counseling from their health care team. Providers were open to implementing a PA-focused RPM solution that efficiently integrated into their workflow and aligned with the principles of health equity and accessibility. As noted previously [19], we found that implementation of an EHR-integrated RPM program into the primary care clinical workflow presents multiple challenges, including limited provider knowledge about exercise counseling, limited provider time, and the need to assist patients in properly setting up and managing wearable devices to allow data to integrate into the EHR. The results of this formative work will inform the development of our future pilot study to establish the feasibility and acceptability of this approach.

### Comparison with Previous Work

Providers expressed strong familiarity with well-known medical guidelines for PA and the ability to provide general counseling to patients. For example, providers appeared familiar with the Centers for Disease Control and Prevention PA guidelines suggesting that adults engage in at least 150-300 minutes of moderately intense aerobic PA weekly [24]. However, while individuals with medical comorbidities who cannot achieve these targets are advised to engage in the maximum level of PA possible to improve health outcomes [24], providers expressed low confidence in their ability to tailor exercise recommendations based on patient-specific factors. Patients in this study also agreed with this sentiment. Furthermore, providers believed they did not have the training or expertise to adequately counsel patients on exercise goals. Yet, evidence and expert guidance indicate patient guidance and titration schedules for both sedentary patients and those with medical comorbidities are available [25]. To address this challenge, strategies to provide succinct evidence-based educational materials with guidance on implementing tailored PA recommendations using remote data should be explored. As providers may be hesitant to increase activity too quickly for the patient with certain medical conditions, these materials can include tailored titration schedules using daily step counts that address this patient’s activity (ie, sedentary lifestyle) and major medical conditions (ie, musculoskeletal conditions, chronic pain, and cardiopulmonary disease) that impact activity guidance. These materials should be made accessible to providers before the implementation of any PA intervention in an electronic format. In addition, patient message templates can be created that include these guidelines that providers can easily elect to send to selected patients through the EHR or standard mail. Future studies should assess the format, delivery mode, and effectiveness of such educational materials in increasing providers’ PA knowledge and tailored PA counseling.

Patients expressed a positive sentiment toward sharing RPM exercise metrics with their provider if this would lead to more specific PA counseling from their health care team. In addition, a subset of our interview sample reported previous experiences with wearable devices that were generally positive but were limited by inconsistent long-term adherence. The effectiveness of mobile health (mHealth) interventions is commonly limited by low long-term adherence rates. For instance, a review of mHealth interventions for anxiety and depression found attrition rates between 1% and 50% [26]. The supportive accountability model proposes that human support increases adherence from an individual who is trustworthy, is benevolent, and has expertise [27]. Our proposed integration of an RPM program within primary care suggests the ability to leverage the provider-patient therapeutic relationship to increase patient adherence.

Providers also expressed concerns about their EHR workflow being overburdened by notifications related to an RPM program. Information overload in the era of EHRs is an important concern. While a small study found physicians are spending about 20% of their time solely on EHR-related tasks [28], studies examining the amount of time PCPs spend reviewing remote data were not readily identified. Information overload specifically related to clinical decision-making systems has been well studied. Repeated similar alerts for the same patient are known to lead to alert fatigue and may lead to lower attention to the alert for its intended function in the primary care setting [29]. While notification fatigue in this practice of primary care is unlikely to be eliminated, we propose measures to limit provider burden using focused in-basket notifications. This may include a provider-initiated limit on notification frequency and an option to receive notifications when data falls outside of prespecified boundaries.

Ultimately, as provider time continues to be burdened by administrative tasks, future studies may need to assess the inclusion of a dedicated team member with specific expertise in exercise counseling for RPM management. A recent study found primary care teams using mHealth strategies can be effective in the care of chronic diseases, such as diabetes [30], suggesting a team-based approach could potentially be effective for lifestyle counseling in primary care. Nurse practitioners have also been suggested as being critical in training, delivering, and monitoring digital PA programs [31]. A dedicated team member for exercise counseling would facilitate increased review of RPM data and allow for dedicated time for communication with patients directly about any challenges with exercise titration potentially due to existing health conditions (ie, chronic pain exacerbation).

Patients express varying comfort with digital technologies, and we identified a major need for patient assistance with device set up and maintenance. Furthermore, this challenge may be intertwined with provider concerns related to health equity and accessibility. Previous studies have noted that access to electronic health portals may be affected by patient factors including age, race, and ethnicity [32]. In our study, patients expressed comfort with written materials as a primary resource for instruction, while also noting access to live support would be helpful. We propose that any RPM protocol should create comprehensive written material for device set up and connection to the medical record. Furthermore, a team member should be available for support on an as-needed basis to both minimize provider burden and assist a diverse array of patients. For the deployment of an RPM program in the real world, we propose a health care team member, such as a medical assistant, should be trained in supporting patients with digital technologies.

Finally, to facilitate the scalability of any RPM program within the clinical setting, insurance reimbursement for the time and decision-making related to RPM is vital. In 2019, the Centers for Medicare and Medicaid Services implemented new billing codes that cover remote monitoring of physiological data, which have also been adopted by some commercial payers. Specific current procedural terminology code examples include billing for services such as device setup, collection or interpretation of physiological data by health care professionals, time-based billing for physicians for communication with patients, and delivery of results by practice staff to the physician [33]. These developing reimbursement mechanisms suggest a potential billing structure to facilitate RPM in the primary care setting.

### Strengths and Limitations

Our study is limited by the fact that we recruited patients and providers from a single, large, urban, academic primary care practice. A relatively small number of participants were interviewed. However, thematic analysis did show we reached a saturation point with the sample size used, consistent with similar qualitative studies [20]. Our sample was comprised largely of female patient participants, although these demographics are consistent with differences in attitudes and behaviors regarding weight [34]. Furthermore, a diversity of viewpoints from provider participants was obtained by provider type and experience level. The EHR used in the study was developed by Epic Systems and we did not attempt RPM integration with any other EHR vendors. Alternate informatics approaches might be needed with other EHRs. However, the principles obtained from participant interviews in this study will still apply broadly to many primary care practices in the development of specific clinical workflows. Finally, while our proposed workflow incorporates RPM data metrics, patients’ subjective impressions (ie, pain with exercise) are not captured using RPM and alternative strategies may need to be developed to elicit this information.

### Conclusions

Our formative assessment found that a potential implementation of an EHR-based RPM program using commercially available wearables for tailored PA counseling by the primary care team was a well-received intervention by both patients and providers. Consistent with our primary objective, we identified key perceptions, needs, and challenges to the implementation of this protocol and proposed potential solutions. Our ongoing work will use these findings in a pilot study to evaluate the feasibility and acceptability of tailored PA counseling leveraging RPM within a primary care practice.

## Appendix

Multimedia Appendix 1 Checklist for Standards for Reporting Implementation Studies (StaRI) guidelines.

Multimedia Appendix 2 Provider job aid instructions.

Multimedia Appendix 3 Patient device syncing instructions.

Multimedia Appendix 4 Provider interview guide.

Multimedia Appendix 5 Patient interview guide.

## Abbreviations

EHR: electronic health record
mHealth: mobile health
PA: physical activity
PCP: primary care provider
RPM: remote patient monitoring
StaRI: Standards for Reporting Implementation Studies
UMass: University of Massachusetts

## Data Availability

The data sets generated during and/or analyzed during this study are available from the corresponding author on reasonable request.
