# Supplementary material for: A Clinician and Electronic Health Record Wearable Device Intervention to Increase Physical Activity in Patients With Obesity: Formative Qualitative Study
Source: JMIR Form Res. 2024 Sep 2;8:e56962. doi: 10.2196/56962 (PMC11406104; doi:10.2196/56962)
Supplement: Multimedia Appendix 2 [file formative_v8i1e56962_app2.docx]

| **Audience: Physicians, APPS** | **Date published: September 21, 2022** |
| --- | --- |
| **Application(s): EpicCare Ambulatory, myChart** | **Tracking number:1** |

Integration with Apple Health and Google Fit allows MyChart to collect data, such as step count, calorie intake, blood pressure, or blood glucose, from other health and fitness apps and incorporate it into a patient's medical record, providing a more complete picture of the patient's health.

When a clinician orders a flowsheet to collect the information she wants to receive, the patient can authorize the Apple Health app or Google Fit app to share the data with MyChart. After MyChart receives the data, it is automatically sent back to the clinician.

Health data collected through the MyChart mobile apps can be used in many ways to help you manage various patient populations. For example:

- Patients with chronic diseases: Track data for hypertension (blood pressure), obesity (weight), osteoarthritis, dysrhythmias (pulse), and more.
- Healthy patients: Track data such as weight, sleep, water consumption, and steps walked.
- Patients at risk of readmission: Track weight for CHF patients or steps walked for patients who have had hip surgery.

Patients use the Track My Health feature, which appears in MyChart for patients with flowsheet orders, to access their flowsheets and track their progress.

**Order myChart Fitness Device Flowsheet**

From your Visit Toolbar, search for “**myChart Fitness**”


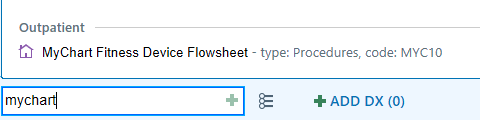


Enter in a Status of Future and the number of days after which you want to be notified about the patient’s flowsheet entries.


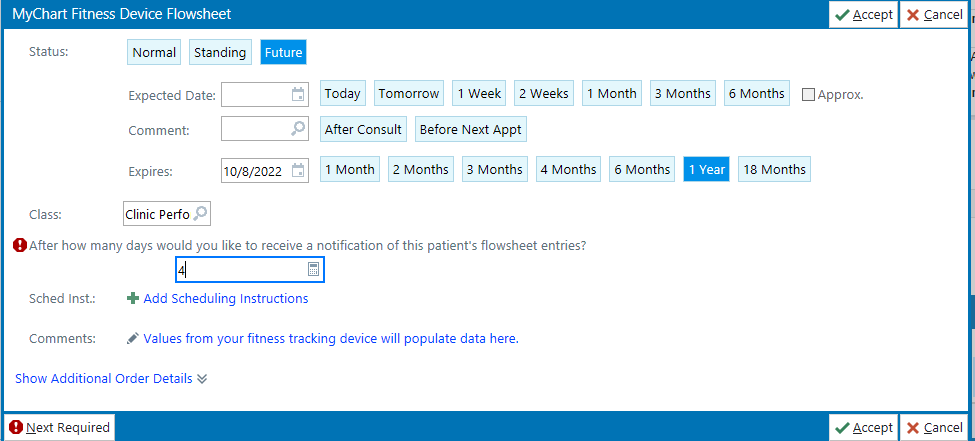


The patient will receive a myChart message indicating a new flowsheet is available. When they click the link in the message, they are brought to the flowsheet where they can connect and manage their devices as well.


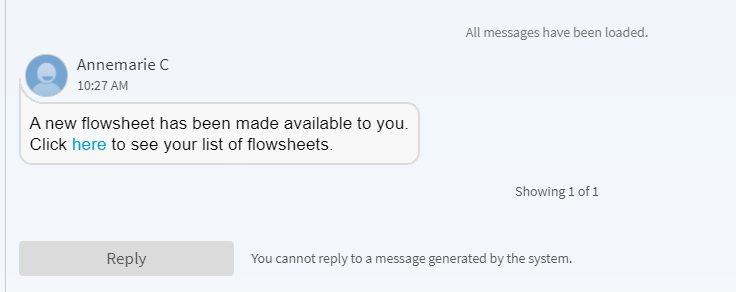

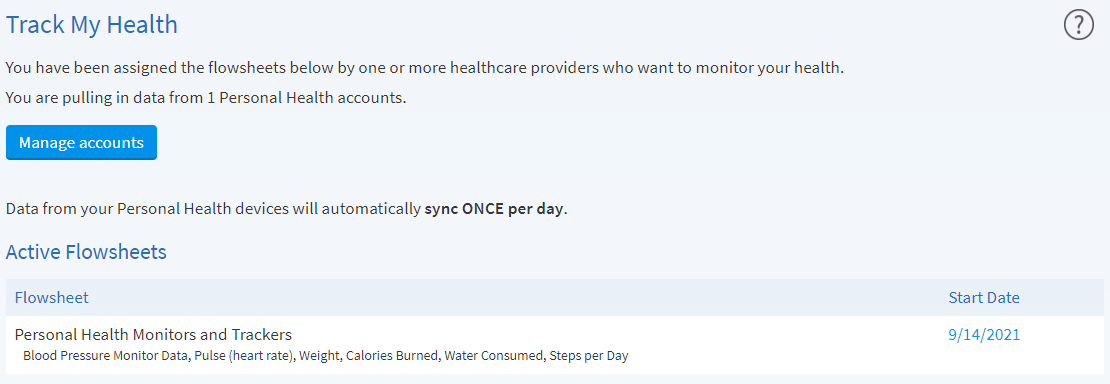


**Where to View Information**

In-Basket

Automated messages can be set up to send pools or providers with a patient’s recent readings, including the ability to send a message after an abnormal reading.


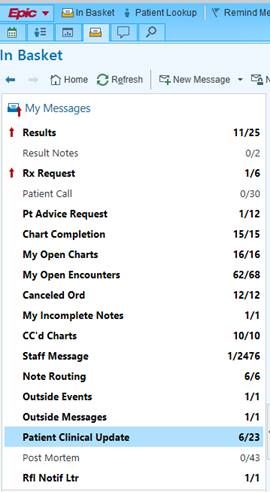


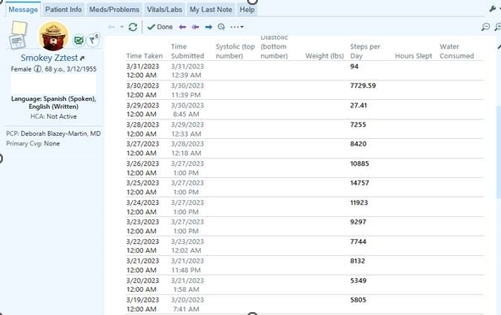


Health Trends via Snapshot

There is a section of Snapshot called Health Trends which will pull the patient-entered data from the patient’s device.


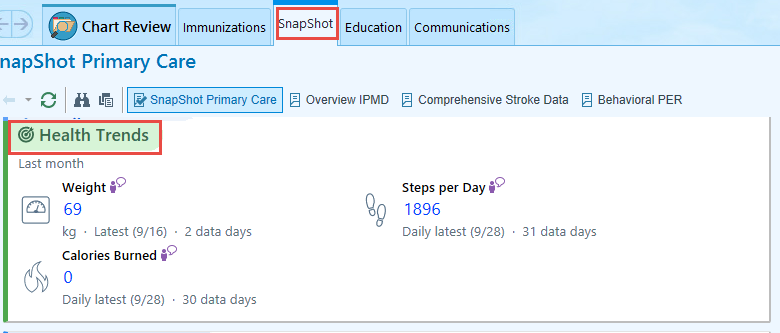


© 2024 Epic Systems Corporation. and The myChart App is powered by MyChart® licensed from Epic Systems Corporation, © 1999 – 2024.
