# Supplementary material for: A Clinician and Electronic Health Record Wearable Device Intervention to Increase Physical Activity in Patients With Obesity: Formative Qualitative Study
Source: JMIR Form Res. 2024 Sep 2;8:e56962. doi: 10.2196/56962 (PMC11406104; doi:10.2196/56962)
Supplement: Multimedia Appendix 3 [file formative_v8i1e56962_app3.docx]

| **Audience: Patients** | **Date published: November 21, 2022** |
| --- | --- |
| **Application(s): myChart** |  |

Do you manage your health using an App or Smartphone? myChart now supports data collected in Apple’s Health app, the Google Fit app, Withings and FitBit!
**Please note: Your provider must place an order for a “myChart Fitness Device” flowsheet to complete this setup.**

**Apple Health**

To sync your myChart app with Apple Health, log into myChart on your Apple device and open the Track My Health feature.

Tap the **Connect to “Health”** button, select which categories you want to allow myChart to update and read and then tap the **Allow** button. We want you to select steps


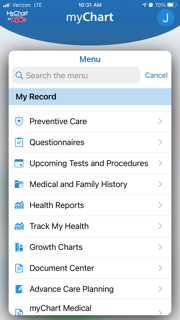

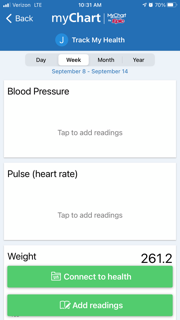

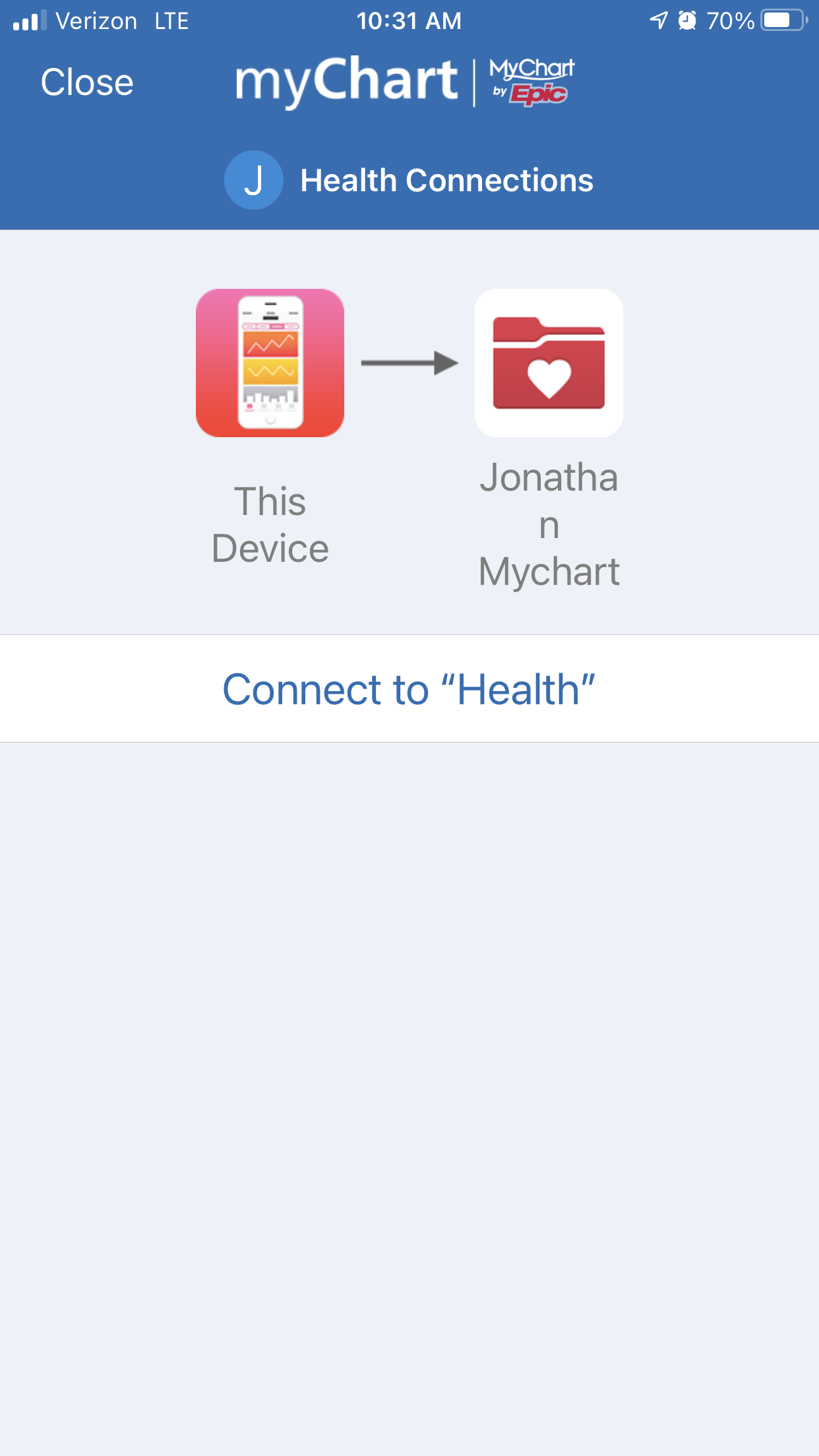

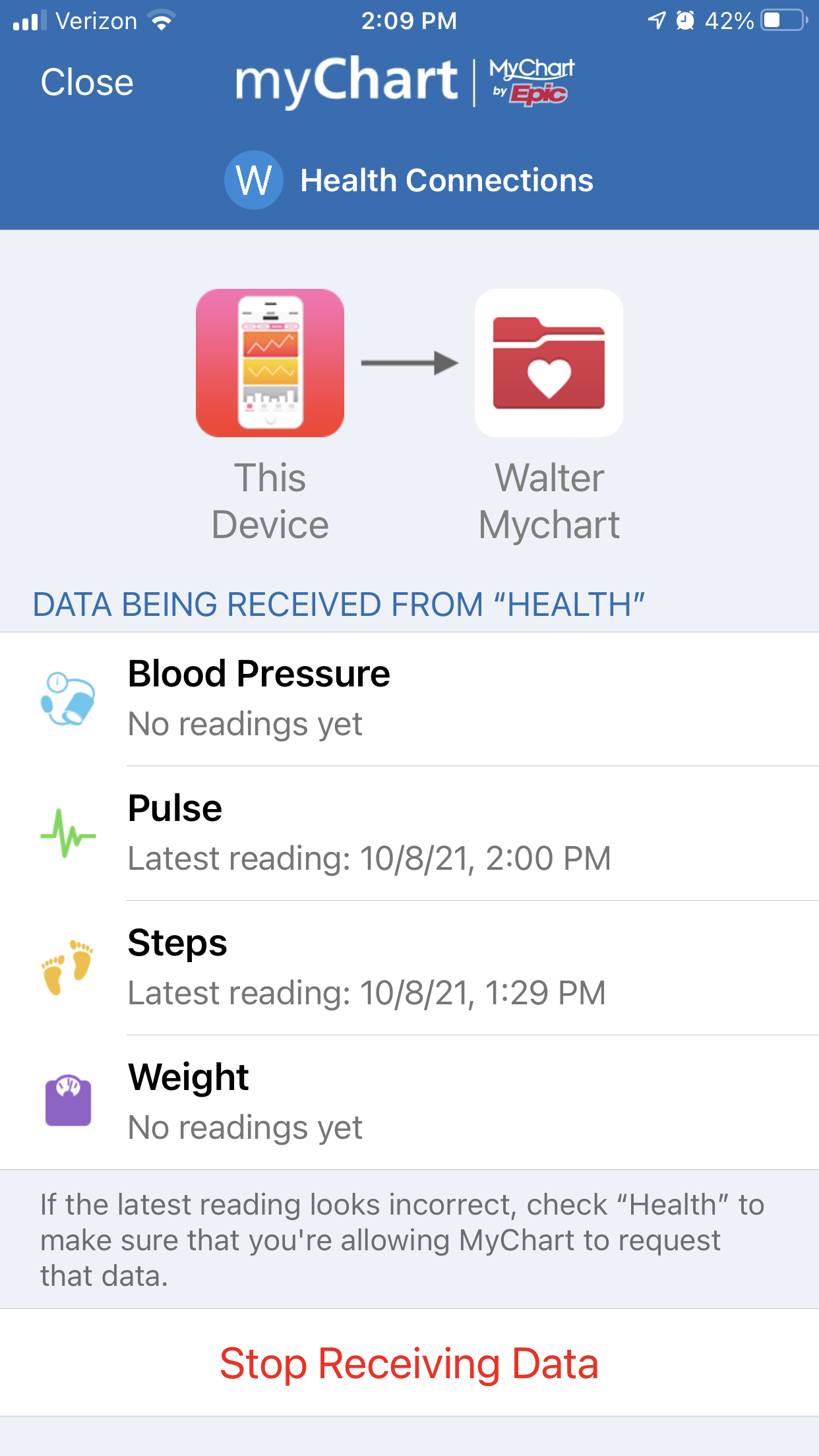


Data will **only** display if your provider has placed the myChart Fitness Device order. Once the steps above have been completed, your data will automatically sync and send to your provider.

You may see additional categories like **Sleep** and **Water Consumed**. These must be **manually entered**.

**Google Fit**

To sync your myChart app with Google Fit, log into myChart on your mobile device and open the Track My Health feature.

Tap the **Manage connections** button, then sign into your Google account to connect to Google Fit. Once signed in, you can select which categories you want to allow myChart to update and read. Your data will sync automatically when connected to wifi.


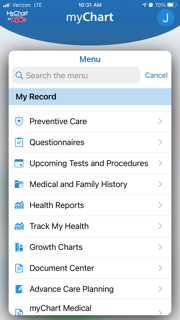

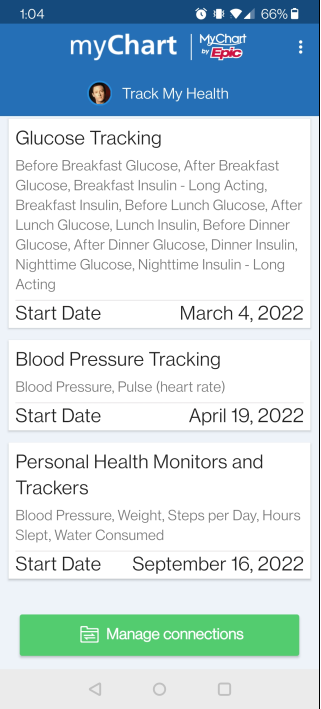

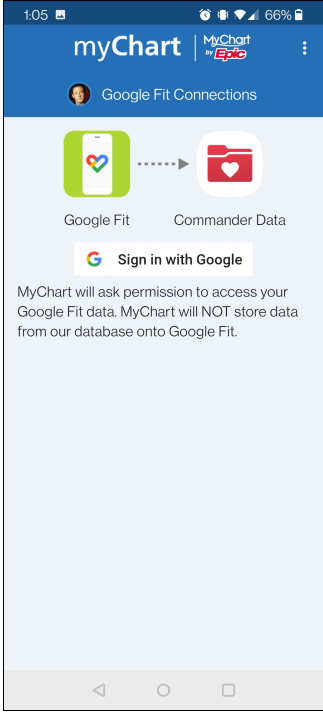

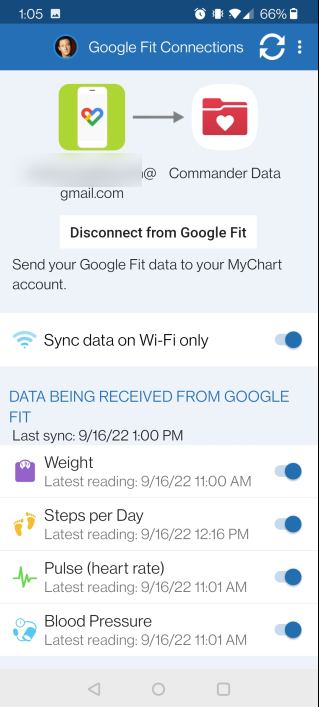


Data will **only** display if your provider has placed the myChart Fitness Device order. Once the steps above have been completed, your data will automatically sync and send to your provider when you are connected to wi-fi.

To manually sync, open the Track My Health feature, tap the **Connect to Google Fit** button, then tap the refresh button at the top right.

**Withings and FitBit**
To sync data from Withings or Fitbit, you must first have an account with the service. Once you have an account, log into myChart and open the **Track My Health** feature.


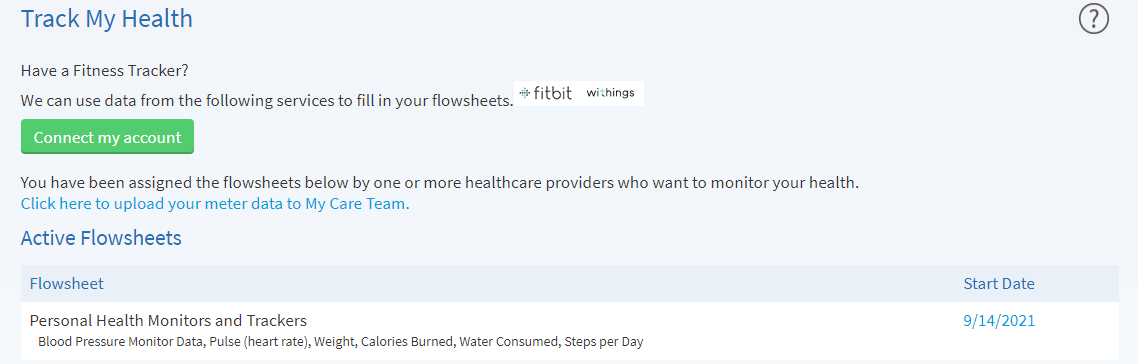


Click on the **Connect My Account** button.


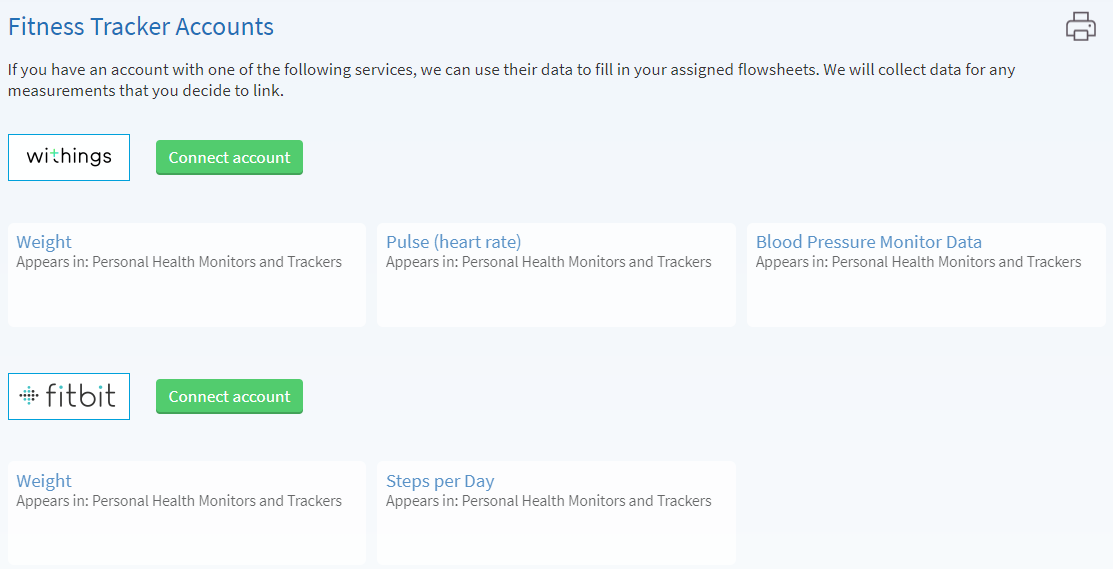


Click on the **Connect Account** button for either Withings or FitBit.

You will be redirected to the website for Withings/FitBit. Click **continue to Withings/FitBit** to continue.


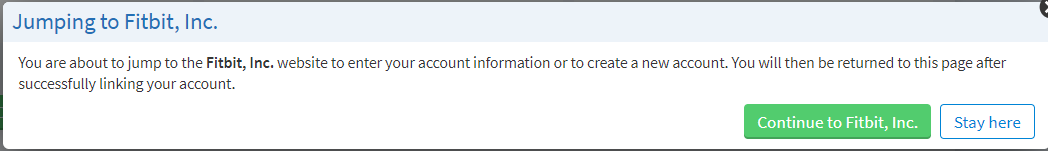


Enter your username and password for Withings/FitBit and sign in.


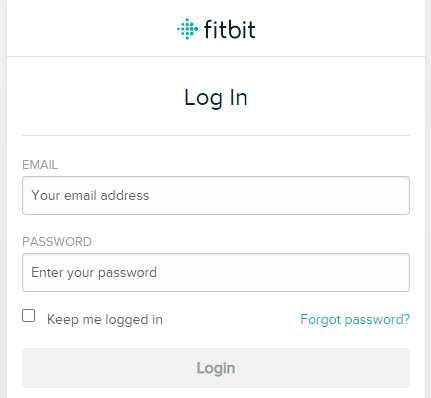


You will be redirected back to myChart. Select the data you want to sync and click the **Start Syncing!** button.


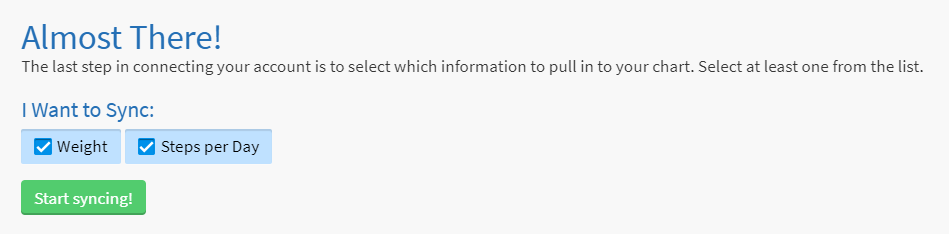


To sync the data from your account with myChart and send it to your healthcare provider, open the fitness tracker page and click the **Sync Now** button.


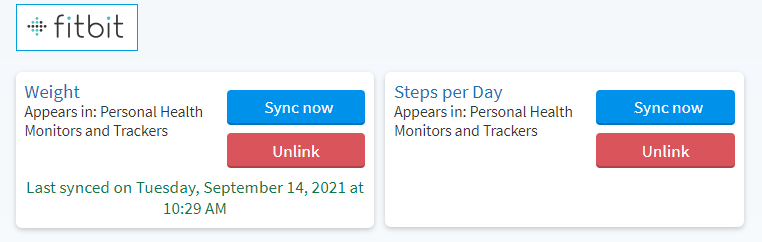


You can unlink your account by clicking the red **Unlink** button. You can review your data anytime by opening the flowsheet in **Track My Health**.

© 2024 Epic Systems Corporation. and The myChart App is powered by MyChart® licensed from Epic Systems Corporation, © 1999 – 2024.
