# Supplementary material for: A Clinician and Electronic Health Record Wearable Device Intervention to Increase Physical Activity in Patients With Obesity: Formative Qualitative Study
Source: JMIR Form Res. 2024 Sep 2;8:e56962. doi: 10.2196/56962 (PMC11406104; doi:10.2196/56962)
Supplement: Multimedia Appendix 4 [file formative_v8i1e56962_app4.docx]

**Provider Interview Guide (Aim 1)**

*Note:* This script and ­­set of instructions are intended as a guide for the interviewer. Interviewer discretion in phrasing, using probes and additional questions or explanation may be necessary because this is a qualitative semi-structured interview script. Qualitative interviews necessarily have a conversational aspect and will almost always diverge at some points from a script. The interviewer may therefore adjust wording, for instance, to acknowledge and take into account that an interviewee has already offered some information in response to a prior question, to clarify a response, or to solicit more information.

Study Overview

*“Our study aims to integrate patient’s wearable device data from a Fitbit into the electronic health record for providers to view and manage. As we develop this protocol, we want to hear your thoughts/perceptions on how we can best integrate this into providers workflow, including identifying and referring patients to the program, then providing feedback on patients’ activity data. Broadly, the study seeks to use exercise as prescription for healthy weight maintenance and overall health”*

1. Overview
   1. What are your current clinical experiences with counseling patients on physical activity?
   2. What is your comfort level in prescribing a specific goal for daily physical activity based on the patients comorbidities, age, and activity level?
      1. What kind of training/support would you need regarding what specific exercise goals are safe and effective for individual patients?
   3. What is your familiarity and opinion of the current use of wearable devices that are directly marketed to patients to track physical activity and other health metrics?
2. Workflow Overview and Feedback
   1. Let’s review the current draft of the clinical workflow for the integration of patient physical activity data. Please ask clarifying questions as I go through it.
   2. What is your opinion of this workflow?
   3. The screenshots I shared with you will serve as the basis of a job aid to assist providers. With the assistance of this job aid, how would you feel about ordering the myChart Fitness Device Flowsheet to integrate patient wearable data into the EMR through mychart?
   4. What would be the best way to receive this data into the EHR for the healthcare team to review? Examples may include In basket messages or message to staff for screening and escalation to provider if needed.
3. Data Utilization
   1. How often do you feel the healthcare team should review patient physical activity data and communicate physical activity recommendation and support to patients?
      1. Should these communication occur through mychart, telephone calls, virtual visits, or in person clinic visit?
   2. If data review and patient counseling was reimbursable how would this change your likelihood of using this type of program?
4. Implementation Support
   1. What kind of support do you/your patients need maximize the clinical benefit and minimize provider burden in this type of program?
      1. Who do you want to provide this support? (Current team members, other staff, health coaches etc.)
   2. What are your concerns about the implementation of a remote monitoring program with patients with obesity to increase physical activity? Examples might include inbox overflow, patient questions, limited clinical utility)
   3. What are your thoughts on digital medicine interventions such as this one being managed by a team led by physicians who are separate from the patients primary care team?

Additional Questions:

1. Health Benefit/Clinical Utility
   1. What are your thoughts on the health benefits of collecting commercial wearable device physical activity data through the medical record?
      1. What would you do with the collected data?
      2. How would this data change your clinical management in assisting patients with health weight management and increasing physical activity?
   2. What commercial activity data would be most useful to you? Examples include Steps/day, Minutes of physical activity per day, and Other non-exercise metrics.
2. Patient Identification
   1. How do you think patients should be identified and selected for inclusion into a physical activity tracking program?
   2. How do you think this option will appeal to diverse patient populations? (Examples could include age and socioeconomic status)
