# Supplementary material for: A Clinician and Electronic Health Record Wearable Device Intervention to Increase Physical Activity in Patients With Obesity: Formative Qualitative Study
Source: JMIR Form Res. 2024 Sep 2;8:e56962. doi: 10.2196/56962 (PMC11406104; doi:10.2196/56962)
Supplement: Multimedia Appendix 5 [file formative_v8i1e56962_app5.docx]

**Patient Interview Guide (Aim 1)**

*Note:* This script and set of instructions are intended as a guide for the interviewer. Interviewer discretion in phrasing, using probes and additional questions or explanation may be necessary because this is a qualitative semi-structured interview script. Qualitative interviews necessarily have a conversational aspect and will almost always diverge at some points from a script. The interviewer may therefore adjust wording, for instance, to acknowledge and take into account that an interviewee has already offered some information in response to a prior question, to clarify a response, or to solicit more information. After you consent the patient:

*“This study will provide patients with a wearable device, or a Fitbit wrist watch, to collect the patient’s physical activity and then enter it right into the electronic health record. As we develop this protocol, we want to hear your thoughts/perceptions on how we can best integrate this into your care, including how we can best support patients to enroll in this program and receive support for activity over the long-term.”*

Overall physical activity

1. What information has your provider discussed about physical activity?
   1. How else do you get your physical activity guidance/advice?
2. What kinds of information would you like on physical activity guidance? Examples include:
   1. How many steps to take per day, minutes of activity with elevated heart rates, etc.
   2. Types of activities to do
   3. Where, when, how long
3. And how would you like to receive any additional information of physical activity?
   1. Format – Paper handout, online…
   2. Delivered from whom?
4. Where do you feel comfortable going for a walk or performing exercise?

Wearable-specific

1. What are your experiences and comfort level with using wearable devices like Fitbit/Apple Watch?
2. What are your thoughts on having your physical activity, like step counts, shared directly with your healthcare team?
3. What do you feel are the most important measurements of physical activity for you and your provider to see?
   1. Examples: Step counts, Minutes in light/moderate/vigorous activity, Minutes of sedentary times, Heart rate during activity
4. Wearable devices work by using an app on your phone to track activity. How would you feel if we were to ask you to download a 2^nd^ app to transfer the data from the app to myChart?
   1. And if you don’t already have myChart, we would ask you to download that as well, resulting in 3 apps. What are your feelings on that process?
5. What support (if any) would you want to setup a wearable device account and connect it to your myChart account?
   1. Example: Support options could include written instructions, video-based instructions, telephone call, zoom call, or in-person support.
6. What guidance (if any) do you want regarding your physical activity and goals?
   1. How frequently would you want to discuss your physical activity?
   2. How would you like to communicate regarding your physical activity and goals? (eg: phone call, my chart message, text message, virtual visit, in-person visit?
   3. Do you have any preferences as to what members of the healthcare team you would like to receive communication from? (ie physicians, NPs/PAs, Nurse, dedicated health coach, medical assistants, clinic staff)
7. What are your thoughts on utilizing youtube or apps to find exercises?
